# Change in emotional distress, anxiety, depression and PTSD from pre- to post-flood exposure in women residing in low-income settings in South Africa

**DOI:** 10.1007/s00737-023-01384-3

**Published:** 2023-11-22

**Authors:** J. Nöthling, A. Gibbs, L. Washington, S. G. Gigaba, S. Willan, N. Abrahams, R. Jewkes

**Affiliations:** 1 Gender and Health Research Unit, South African Medical Research Council, Francie van Zijl DriveTygerberg, PO Box 19070, Cape Town, 7505 South Africa; 2 Department of Psychology, University of Exeter, Exeter, UK; 3 Centre for Rural Health, University of KwaZulu-Natal, Durban, South Africa; 4 Institute for Global Health, University College London, London, UK; 5 Project Empower, Durban, South Africa; 6 The School of Applied Human Sciences (Psychology), University of KwaZulu-Natal, Durban, South Africa; 7 School of Public Health and Family Medicine: Faculty of Health Sciences, University of Cape Town, Cape Town, South Africa; 8 School of Public Health, Faculty of Health Sciences, University of the Witwatersrand, Johannesburg, South Africa

**Keywords:** Floods, Emotional distress, Depression, Posttraumatic stress disorder, Anxiety

## Abstract

Floods are increasing in frequency and may increase the risk for experiencing emotional distress, anxiety, depression and PTSD. The aim of this study was to determine the extent of damage, loss, injury and death resulting from floods that occurred in and around the city of Durban, South Africa, in April 2022, and associated changes in mental health pre- to post-floods in a low-income setting. Seventy-three women between the ages of 18 and 45, residing in flood affected, low-income settings, were interviewed prior to the floods occurring. Mental health measures were repeated with 69 of the 73 women during the post-flood interview along with a questionnaire measuring flood-related exposures. Loss of infrastructure (lacked access to drinking water, electricity, fresh food, could not travel to work, had to stay in a shelter and could not get hold of friends or family) was a predictor of post-flood change in levels of emotional distress and anxiety. Higher levels of prior trauma exposure were associated with higher post-flood levels of emotional distress. Higher pre-flood food insecurity was also associated with higher post-flood anxiety. Women affected by poverty, food insecurity and a history of trauma are vulnerable to the additive adverse mental health effects of floods. Proactive approaches to diminishing the impact of floods on the livelihood of women is needed and post-flood relieve efforts may be more affective if they are enhanced by providing mental health support.

**Supplementary Information:**

The online version contains supplementary material available at 10.1007/s00737-023-01384-3.

## Introduction

Regional changes in the water cycle, as a result of climate change, has brought about an increase in natural disasters and in particular the frequency of flooding (Eccles et al. 2019; McClymont et al. 2020). According to the Emergency Event Database (EM-DAT) which records and stores information on disasters across the globe, floods accounted for 51.6% of natural disasters in 2021 (Guha-Sapir et al. 2021). In the same period, floods affected 29.2 million individuals and resulted in $74.4 billion in economic loss and 4143 lives lost (Guha-Sapir et al. 2021). In addition to the immediate health effects of floods (e.g. death and injury), floods may have indirect long-lasting health effects such as an increase in vector-borne diseases (e.g. malaria, Rift Valley fever and West Nile fever) and water-borne diseases (e.g. gastroenteritis, cholera, typhoid fever and dysentery) (Chowell et al. 2019; De Souza et al. 2015; Fahy et al. 2019; Suhr and Steinert 2022). The loss of cattle, crops and reduced soil quality following floods also has an impact on food security which may result in hunger and malnutrition (Codjoe and Owusu 2011; Hathaway and Maibach 2018; Opoku et al. 2021).

Another indirect impact of floods includes emotional and psychological difficulties resulting from flood-related traumatic experiences (Fernandez et al. 2015). These traumatic experiences includes displacement, loss of life, loss of belongings as well as loss of community cohesion and support (Bandla et al. 2019; Fernandez et al. 2015). Flood-related trauma increases the risk for post-traumatic stress disorder (PTSD), depression and anxiety (Cianconi et al. 2020). A study conducted in informal settlements in Kenya found that 80.8% of participants experienced an increase in psychological distress in the immediate aftermath of floods (Okaka and Odhiambo 2019). A recent systematic review and meta-analysis of 23 studies (predominately studies from low- and middle-income countries—LMIC) on the development of PTSD following floods, reported a prevalence rate of 29.5% which is considerably higher than the estimated global prevalence of 3.9% (Golitaleb et al. 2022; Koenen et al. 2017). Depression and anxiety following flood exposure is less commonly studied, but the prevalence is expected to be high given that these conditions are highly comorbid with PTSD (Cianconi et al. 2020).

The adverse psychological effects of floods are often correlated with the severity of the impact of floods e.g. more losses, damage, threat and harm experienced results in more psychological distress, and these effects are more pronounced in LMIC’s characterized by high levels of poverty and food insecurity (Bouchard et al. 2022; Frumkin et al. 2008; Opoku et al. 2021). The mental health effects of floods is further exacerbated by prior experiences of psychological symptoms and prior exposure to traumas (e.g. childhood trauma and gender-based violence) (Alderman et al. 2012; Cianconi et al. 2020; Fernandez et al. 2015; Stanke et al. 2012). Women, children, elderly and those with disabilities are often disproportionally affected by poverty and trauma which may place them at higher risk for adverse mental health outcomes following floods (Fahy et al. 2019; Gruebner et al. 2017; Hetherington et al. 2018; Veenema et al. 2017). The psychological effects of floods are generally less often reported than economic loss and injuries, especially in LMICs (Sohrabizadeh et al. 2016). This results in an incomplete picture of the need for health and mental health services in flood affected regions, which adversely affects resource allocation and service delivery planning (Bouchard et al. 2022; Elwenspoek et al. 2020; Fernandez et al. 2015).

An opportunity to study the mental health impacts of floods was presented when severe flooding occurred between 8 and 12 April 2022 in the city of Durban and surroundings, located in the eThekwini municipality of South Africa (South African Weather Service 2022). Flooding in this region is not uncommon with some flood-related damage reported yearly and more severe flooding events, which includes loss of live, reported once or twice during a decade (Olanrewaju & Reddy, 2022). However, during the April 2022 floods record-breaking rainfall was recorded, with some areas reporting more than 300 mm of rain in 24 h (South African Weather Service 2022). The heavy rains, already saturated ground and inadequate draining systems brought about flash floods and landslides which resulted in more than 450 deaths, destruction of 8584 houses and severe damaged to 13,536 houses, 600 schools and 84 health care facilities (International Federation of the Red Cross 2022). Some of the damage was also a result of the overflowing of several rivers in the area (International Federation of the Red Cross 2022). Electricity, road and water infrastructure were severely damaged resulting in loss of electricity, access to drinking water, cellphone signal and food supplies, lasting for more than a week in the most severely affected areas (Bouchard et al. 2022; International Federation of the Red Cross 2022; South African Weather Service 2022). Collapsed roads and destroyed bridges delayed rescue operations and access to health care services (International Federation of the Red Cross 2022). The worst household flooding was reported before sunrise in the early hours of the morning of the 12^th^ of April which, in addition to the electricity outages, complicated residential evacuation procedures (Bouchard et al. 2022). The total cost of infrastructure damaged caused by the floods is estimated to be around R25 billion (US$1.5 billion) and recovery operations and repairs are still in progress (International Federation of the Red Cross 2022).

The natural landscape in the eThekwini municipal region is characterized by rolling hills, valleys and streams (Diop et al. 2010). Informal settlements in this region are growing in size due to regional migration and urbanization (Bouchard et al. 2022). Housing in informal settlements is built from a mixture of materials, with brick or concrete structures interspersed with less durable structures made from zinc-electroplated corrugated iron (known as ‘zinc’) sheets and (often reused) wood, the roof of which, at the best of times, often leak (International Federation of the Red Cross 2022). The informal settlements are commonly near rivers, below flood lines or at the foot of steep hills (Williams et al. 2019). High levels of poverty, unemployment and overcrowding, and inadequate access to drinking water, sanitation services, drainage, waste disposal and health services are also frequently reported (Bouchard et al. 2022; Cianconi et al. 2020; Hlahla et al. 2022). Deforestation and substandard housing development practices, coupled with the landscape characteristics and heavy rains, left the residents of these areas particularly vulnerable to the damage caused by the April floods (Williams et al. 2019). Landslides were widespread and resulted in injuries and death caused by colliding with debris, entrapment and suffocation (Diop et al. 2010).

At the time of the floods, we had commenced interviews with a group of women from flood-affected areas. We adapted our study, as described below, in order to investigate the impacts of the floods on their wellbeing. In this paper we consider three questions: first, what was the extent of damage, loss, injury and death related to the floods, reported by women living in flood-affected low-income settings? Second, what were the associations between flood impact (damage to belongings, loss of infrastructure and interpersonal impact) and pre- to post-flood symptoms of general emotional distress, depression, anxiety and PTSD in this group of women? Third, did experiences of childhood trauma, intimate partner violence (IPV), non-partner sexual violence (NPSV) and food insecurity have an impact on symptoms of emotional distress, depression, anxiety and PTSD pre- and post-floods?

## Methods

### Participants and setting

The original aim of the broader study was to conduct one set of interviews with women to assess standard measures for future use in a randomised control trial called the Rape Impact Cohort Evaluation Study 2 (registered in the Pan African Clinical Trail Registry under PACTR202211795963218). In order to do this, 100 women between the ages of 18 and 45 were recruited (using convenience sampling) from low-income settings (predominately informal settlements) in the eThekwini district through a local non-governmental organisation (NGO) Project Empower, that worked in these areas. These interviews were conducted between 29 March 2022 and 25 April 2022 with 100 women, however, prior to the occurrence of the floods on 8 April 2022, 73 of these women had been interviewed. The 73 women were invited to return to the study site to complete a follow-up questionnaire between 20 June 2022 and 13 July 2022 which was between two and three months after the floods occurred. Sixty-nine women returned and completed the follow-up questionnaire.

### Procedure

The original study with the 100 participants was introduced as the ‘Women’s Health and Wellbeing Study’ and the procedures were briefly explained telephonically. Interested participants were invited to attend an information session at the site of the NGO’s offices where they also completed the informed consent procedures before enrolling in the study. Participants were informed that participation is voluntary and that they could withdraw from the study at any time. After explaining the study procedures and obtaining informed consent, participants were invited to complete the pre-flood assessments with a research assistant who was trained in ethical principles in research and working with vulnerable groups. The assessments were completed using a one-on-one interview format where the research assistant read each question to the participant and recorded the responses. An assessment lasted approximately one hour and consisted of a demographic questionnaire and self-report measures. Participants were offered a break if the research assistant noticed that they were becomming restless or fatigued. The subgroup of women who returned for the nested flood study (69) were reconsented and informed of the amended aim of the study which was to compare pre- and post-flood mental health outcomes. Self-report measures related to mental health outcomes were readministered along with a measure assessing the severity of exposure to flood-related damage, loss, injury and death.

 Women were referred to a local university’s trauma clinic (operated by trainee clinical and counselling psychologists) if they exhibited signs of distress or indicated a need for support or specialised care. All data gathered were deidentified by replacing identifiable information with a study number. Women were compensated for their time and travel expenses. Ethical approval to conduct the original and follow-up study was obtained from the South African Medical Research Council Ethics Committee (SAMRC; EC041-10/2021).

### Measures

Demographic data were collected along with mental health data which included a general emotional distress, depression, anxiety and PTSD measure. Potential mental health covariates measured included food insecurity, childhood trauma, IPV and NPSV exposures. The impact of the floods was measured by inquiring about damage to belongings, loss of infrastructure and, interpersonal impact. The measures used are described in Table 1.

**Table 1:** Description of measures and variables used in the study

Measure	Description	Timepoint/s administered and Cronbach alpha	Variable/s	Source of measure
Demographic questionnaire	Six items with diverse response options depending on individual items	Pre-flood	(1) Age, (2) ethnicity, (3) education, (4) main source of income, (5) amount of monthly income	
Kessler Psychological Distress Scale (K6)	Six items measuring psychological distress related to depression and anxiety e.g. ‘during the past 30 days, how often did you feel hopeless?’ Response options were adjusted for consistency with other scales and were rated on a 4-point Likert scale ranging from 0 ‘not at all’ to 4 ‘almost always’. Total scores raged between 0 and 24. Higher scores represented higher psychological distress.	Pre- (0.84) and post-flood (0.89)	Emotional distress	(Kessler et al. 2002)
Centre for Epidemiologic Studies Depression Scale (CES-D)	Twenty items measuring symptoms of depression e.g. ‘I felt that I could not shake off the blues even with help from my family or friends’. Responses were recorded on a 4-point Likert scale ranging from 0 ‘rarely or none of the time’ to 3 ‘most or all of the time’. The total scores ranged between 0 and 60. Higher scores indicated more severe symptoms of depression. A cut-off score of 21 or more was considered indicative of possible depression.	Pre- (0.80) and post-flood (0.89)	Depression symptoms and status	(Radloff 1977)
Generalised Anxiety Disorder 7 (GAD7)	Seven items assessing symptoms of anxiety e.g. ‘during the past two weeks, how often did you have trouble relaxing?’. Responses were recorded on a 4-point Likert scale ranging from 0 ‘not at all’ to 3 ‘nearly every day’. Total scores ranged between 0 and 21. Higher scores represented higher anxiety levels. A cut-off of 10 indicated moderate anxiety.	Pre- (0.85) and post-flood (0.88)	Anxiety symptoms and status	(Spitzer et al. 2006)
Harvard Trauma Questionnaire (HTQ)	Sixteen items measuring DSM-IV symptoms of PTSD experienced in the past week e.g. ‘nightmares about the event keep coming back’. Responses were recorded on a 4-point Likert scale ranging from 1 ‘never’ to 4 ‘often’. Total scores ranged between 16 and 64. Higher scores indicated more severe PTSD symptoms. A cut-off score was calculated by dividing the total score by 16, with those scoring above 2.5 being considered as possible PTSD cases.	Pre- (0.91) and post-flood (0.92)	PTSD symptom severity and status	(Mollica et al. 1992)
Household Hunger Scale (HHS)	Three items enquired about the frequency of lack of food in the household, going to sleep hungry and going a whole day without eating due to a lack of food. Responses were recorded on a 4-point Likert scale ranging from 1 ‘never’ to 3 ‘often’. Total scores ranged between 3 and 12. Higher scores indicated more severe hunger.	Pre-flood (0.85)	Food insecurity	(Deitchler et al. 2010)
Childhood Trauma Questionnaire – Short Form (CTQ-SF)	A modified version of the CTQ-SF was used to measure exposure to neglect, emotional abuse, physical abuse and sexual abuse before the age of 18. The measure consisted of fourteen items e.g. ‘I was beaten so hard at home that it left a mark or bruise’ with responses measured on a 4-point Likert scale. For each item participants were asked to indicate if the trauma occurred 1 ‘never’, 2 ‘rarely’, 3 ‘sometimes’ or 4 ‘often’. Total scores ranged between 14 and 56 with higher scores indicating more severe exposure. Responding ‘rarely’, ‘sometimes’ or ‘often’ to any of one the items measuring neglect, emotional abuse, sexual abuse or physical abuse, respectively, was considered indicative of the presence of these types of childhood trauma.	Pre-flood (0.68)	Childhood neglect, emotional abuse, sexual abuse and physical abuse	(Bernstein et al. 2003)
Intimate Partner Violence (IPV) and Non-Partner Sexual Violence (NPSV)	IPV was measured using five items enquiring about physical IPV e.g. ‘in the past 6 months, how many times has your current or previous husband or boyfriend kicked, dragged, beaten, choked or burnt you? and three items enquiring about sexual IPV e.g. ‘in the past 6 months, how many times has your current or previous husband or boyfriend physically forced you to have sex when you did not want to?’. NPSV was measured using four items enquiring about past experiences of non-partner rape e.g. ‘in the past 6 months, how many times have you been forced or persuaded to have sex against your will by a man who wasn’t your husband or boyfriend?’. Response options included 1 ‘never’, 2 ‘once’, 3 ‘a few times’ and 4 ‘many times’. Responding ‘once’, ‘a few times’ or ‘many times’ to any one of the items measuring physical IPV, sexual IPV and NPSV, respectively, was considered indicative of the presence of these types of violence in the past 6 months.	Pre-flood (nominal measure)	Physical IPV, sexual IPV and NPSV	(García-Moreno et al. 2006)
Composite Trauma Load Score	A composite trauma score was calculated by assigning a score of 1 to each trauma type if present or 0 if not present. The trauma types included childhood neglect, childhood emotional abuse, childhood sexual abuse, childhood physical abuse, physical IPV, sexual IPV and NPSV. The composite score represented the number of different trauma types a participant was exposed to. Scores ranged between 0 and 7.	Pre-flood (ordinal measure)	Trauma exposure	
Flood Impact Questionnaire	Four items measured damage to belongings (furniture, electrical appliances, cell phone or computer, house structure) with responses measured on a 3-point Likert scale with response options 0 ‘no damage’, 1 ‘some things were damaged’ and 2 ‘everything was destroyed or lost’. Six items measured loss of infrastructure (lacked access to drinking water, electricity, fresh food, travel to work, had to stay in a shelter and could not get hold of others). Responses were measured on a 4-point Likert scale with response options 0 ‘not true at all’, 1 ‘for 1 to 7 days’, 2 ‘for 8 to 14 days’ and 3 ‘more than 15 days’. Six items measured interpersonal impact (significant other still missing, significant other passed away, significant other was injured, witnessed someone passing away, witnessed someone getting injured, had to take care of someone who lost their home). Responses were measured on a 3-point Likert scale with response options 0 ‘no’, 1 ‘yes, 1 person’, 2 ‘yes, more than 1 person’. The items were newly developed, based on the literature reporting the experiences and impact of the April 2022 floods on the eThekwini population (Bouchard et al., 2022; International Federation of the Red Cross 2022). Higher scores indicated being more severely impacted by the floods.	Post-flood (0.64-0.77)	Effect of floods on the individuals, categorised as damage to belongings, loss of infrastructure and interpersonal impact	

### Data analysis

Data analysis was completed using SPSS version 27 and Stata version 17. Baseline demographic and psychological characteristics of women who completed the pre-and post-flood assessments were compared to women who only completed the pre-flood assessment, to assess for selection bias, using chi-square and t-test statistics. Descriptive statistics (mean, standard deviation, percentages) were calculated for the demographic and psychosocial variables, flood-related damage to belongings, loss of infrastructure and interpersonal impact. Change in PTSD, depression, anxiety and emotional distress scores from pre- to post-flood assessment were analysed using paired sample t-tests for continuous outcomes and McNemar’s exact tests for binary outcomes (cut-off scores). Pearson’s correlation coefficients were calculated to assess the relationship between flood-related damage to belongings, loss of infrastructure, interpersonal impact and post-flood scores for emotional distress, depression, anxiety and PTSD. A composite pre-flood trauma score (childhood trauma, IPV and NPSV experiences), food insecurity scores and pre- and post-flood scores for emotional distress, depression, anxiety and PTSD was also investigated using Pearson’s correlation coefficients.

Four linear regression models were used to assess whether there was an independent association between the impact of damage to belongings, loss of infrastructure and interpersonal impact on post-flood levels of emotional distress (model 1, controlling for baseline emotional distress and age), depression (model 2, controlling for baseline depression and age), anxiety (model 3, controlling for baseline anxiety and age) and PTSD (model 4, controlling for baseline PTSD and age). Structural equation modelling (SEM) was used to assess the relationships between the flood-related variables that were significantly associated with the mental health outcomes in the regression models. We also included pre-flood prior trauma and food insecurity in the SEM models as covariates. Associations were tested using the maximum likelihood method. Goodness of fit was assess using a comparative fit index (CFI) of > 0.950, a Tucker-Lewis index of > 0.950 and a root mean square error of approximation (RMSEA) of < 0.050.

## Results

### Baseline demographic, food insecurity and prior trauma characteristics of the sample

There were no significant differences in baseline psychosocial characteristics between women who completed the pre-flood assessment and those who completed the pre- and post-flood assessment (see supplementary Table 1). Table 2 present the baseline socio-demographic characteristics of the sample. The mean age of participants was 26.3 years. All participants completed some form of education with 58% indicating completion of primary education, 40.6% indicating completion of secondary education and 1.4% indicating completion of tertiary education. The majority of participants were unemployed with 71% relying on social grants as a primary source of income. None of the participants indicated that they had no money available for expenses per month, 42% indicated that they had between R1 and R500 ($0 – $31) available followed by 30.6% who had between R501 and R1000 ($31 – $61) available, 13% who had between R1001 and R2000 ($61—$122) available and 2.9% ($122) had more than R2001 available. The mean score for food insecurity was 4.6 (range 3–12). The mean composite childhood, IPV and NPSV pre-flood trauma load score was 2.9 (range 0–7) with the most prominent trauma being childhood physical abuse with a prevalence of 69.6%, followed by 55.1% for childhood neglect, 52.2% for childhood emotional abuse, 34.8% for physical IPV, 34.8% for sexual IPV, 29% for childhood sexual abuse and 14.5% for sexual NPSV.

**Table 2:** Demographic, food insecurity, trauma and mental health characteristics of the sample

	*n* (%)	M (SD)	Range
Age	69 (100)	26.3 (5.7)	18–44
Level of education completed	69 (100)		
No formal education	0 (0.0)		
Primary education	40 (58)		
Secondary education	28 (40.6)		
Tertiary education	1 (1.4)		
Main source of income	69 (100)		
No income	1 (1.4)		
Employment	9 (13.)		
Social grant	49 (71)		
Family support	8 (11.6)		
Partner support	5 (7.2)		
Other^1^	1 (1.4)		
Amount of money available to spend per month^2^	68 (100)		
None	0 (0.0)		
R1 – R500 ($0 – $31)	29 (42)		
R501 – R1000 ($31 – $61)	28 (30.6)		
R1001 – R2000 ($61—$122)	9 (13)		
> R2001 (> $122)	2 (2.9)		
Household Hunger Scale (HHS) score	69 (100)	4.6 (2.2)	3–12
Childhood Trauma Scale (CTQ) score	69 (100)	22.2 (6.5)	14–40
Experienced childhood neglect	38 (55.1)		
Experienced childhood emotional abuse	36 (52.2)		
Experienced childhood physical abuse	48 (69.6)		
Experienced childhood sexual abuse	20 (29.0)		
Intimate partner violence (IPV)	69 (100)		
Experienced physical IPV	24 (34.8)		
Experienced sexual IPV	24 (34.8)		
Non-partner violence (NPV)	69 (100)		
Experienced sexual NPV	10 (14.5)		
Composite pre-flood trauma load score	69 (100)	2.9 (1.7)	0–7
Emotional distress (K6) pre-flood	67 (100)	15.9(5.4)	6–24
Emotional distress (K6) post-flood	69 (100)	15.1(5.4)	6–24
Depression (CESD) pre-flood	66 (100)	23.9(12.4)	3–51
Depression (CESD) post-flood	68 (100)	20.3(12.2)	0–44
Anxiety (GAD7) pre-flood	69 (100)	8.4(5.2)	0–21
Anxiety (GAD7) post-flood	69 (100)	8.7(5.4)	0–21
PTSD (HTQ) pre-flood	68 (100)	32.9(13.1)	16–63
PTSD (HTQ) post-flood	69 (100)	35.2(12.4)	16–60

1. Student bursary funding provided by the South African National Student Financial Aid Scheme (NSFAS)

2. Based on the average United States Dollar ($) to South African Rand (ZAR) exchange rate of $1 to R16.4 in 2022

*HHS* Household Hunger scale; *CTQ* Childhood trauma scale; *IPV* Intimate partner violence; *NPV *Non-partner violence; *K6* Kessler Psychological Distress Scale; *CES-D* Centre for Epidemiologic Studies Depression Scale; *GAD7* Generalised Anxiety Disorder 7; *PTSD* Posttraumatic stress disorder; *HTQ* Harvard Trauma Questionnaire

### Pre- and post-flood mental health characteristics of the sample

Table 3 presents the pre- and post-flood mental health symptom scores of the participants. There were no significant differences in pre- to post-flood scores for emotional distress (*p* = 0.247), anxiety (*p* = 0.686) and PTSD (*p* = 0.138). There was a significant decline in pre- (*M* = 23.9, *SD* = 12.4) to post-flood (*M* = 20.3, *SD* = 12.2) depression scores (*p* = 0.036).

**Table 3:** Change in cut-off scores for depression, anxiety and PTSD

		Depression post-flood		*p*
		Above cut-off *n* (%)	Below cut-off *n* (%)	
				0.648
Depression pre-flood	Above cut-off *n* (%)	27 (41.5)	8 (12.3)	
	Below cut-off *n* (%)	11 (16.9)	19 (29.2)	
	Total *n* (%)	38 (58.5)	27 (41.5)	
		Anxiety post-flood		*p*
		Above cut-off *n* (%)	Below cut-off *n* (%)	
				0.189
Anxiety pre-flood	Above cut-off n(%)	16 (23.2)	7 (10.1)	
	Below cut-off n(%)	14 (20.3)	32 (46.4)	
	Total *n* (%)	30 (43.5)	39 (56.5)	
		PTSD post-flood		*p*
		Above cut-off *n* (%)	Below cut-off *n* (%)	
				0.049*
PTSD pre-flood	Above cut-off *n* (%)	13 (19.1)	4 (5.9)	
	Below cut-off *n* (%)	13 (19.1)	38 (55.9)	
	Total *n* (%)	26 (38.2)	42 (61.8)	

*PTSD* Posttraumatic stress disorder

*p < 0.05

When considering pre- and post-flood cut-off scores, the difference between depression groups and anxiety groups was not significant. There was a significant difference between PTSD groups (*p* = 0.049), with 19.1% of participants scoring above cut-off pre- and post-floods, 55.9% scoring below cut-off pre- and post-floods, 19.1% scoring below cut-off pre-floods and above cut-off post-flood, and 5.9% scoring above cut-off pre-floods and below cut-off post-floods.

### Damage, loss, and interpersonal impact of the floods

Flood-related characteristics are presented in Table 4. More than half of the sample reported some damage to furniture (53.6%) and some structural damage to their homes (65.7%), less reported damage to electrical appliances (26.1%) and damage to phones or computers (11.6%).The most common category endorsed for destroyed items was electronic appliances (10.1%) followed by housing structures (7.5%) and furniture (5.8%). In the wake of the floods, the majority of participants (86.9%) lacked access to drinking water and electricity (75.4%) for at least 1 to 7 days while 46.3% did not have access to fresh food, 17.3% could not travel and lost income, 11.5% had to stay in a shelter and 20.2% could not get hold a family member for at least 1 to 7 days. The death of a significant other (family member, friend or someone they cared about) was reported by 15.9% of participants, 21.7% reported an injury of a significant other, 2.8% reported a significant other still being missing and 15.9% reporting having to care for a significant other who lost their home. Witnessing injury caused by the floods was reported by 15.9% of participants and 5.8% witnessed a death.

**Table 4:** Frequency and type of damage, loss resulting from the floods

	Not at all	Some things were damaged	Everything was destroyed or lost		M(SD)	Range
	*n* (%)	*n* (%)	*n* (%)			
Damage to belongings					2.01 (1.69)	0–6
Damage to furniture	28 (40.6)	37 (53.6)	4 (5.8)		0.65 (0.59)	0–2
Damage to electrical appliances	44 (63.8)	18 (26.1)	7 (10.1)		0.46 (0.68)	0–2
Damage to cell phone or computer	61 (88.4)	8 (11.6)	0 (0.0)		0.12 (0.32)	0–1
Damage to house structure	18 (26.9)	44 (65.7)	5 (7.5)		0.81 (0.56)	0–2
	Not true at all	Yes, for 1–7 days	Yes, for 8–14 days	Yes, for more than 15 days		
	*n* (%)	*n* (%)	*n* (%)	*n* (%)		
Loss of infrastructure					4.13 (3.20)	0–14
Did not have access to drinking water	9 (13.0)	33 (47.8)	12 (17.4)	15 (21.7)	1.48 (0.98)	0–3
Did not have access to electricity	17 (24.6)	30 (43.5)	12 (17.4)	10 (14.5)	1.22 (0.98)	0–3
Did not haveed access to fresh food	37 (53.6)	21 (30.4)	7 (10.1)	4 (5.8)	0.68 (0.88)	0–3
Could not travel to work and lost income	57 (82.6)	9 (13.0)	3 (4.3)	0 (0.0)	0.26 (0.68)	0–3
Did not have access to my home and had to stay in a shelter	61 (88.4)	5 (7.2)	1 (1.4)	2 (2.9)	0.19 (0.60)	0–3
Did not have cell phone reception and could not get hold of significant others	55 (79.7)	10 (14.5)	1 (1.4)	3 (4.3)	0.30 (0.71)	0–3
	No	Yes, 1 person	Yes, more than 1 person			
	*n* (%)	*n* (%)	*n* (%)			
Interpersonal impact					1.57 (2.40)	0–9
Significant other still missing	67 (97.1)	1 (1.4)	1 (1.4)		0.01 (0.12)	0–1
Significant other passed away	58 (84.1)	4 (5.8)	7 (10.1)		0.26 (0.63)	0–2
Significant other was physically injured	54 (78.3)	7 (10.1)	8 (11.6)		0.33 (0.68)	0–2
Witnessed someone getting injured	58 (84.1)	7 (10.1)	4 (5.8)		0.22 (0.54)	0–2
Witnessed someone pass away	65 (94.2)	4 (5.8)	0 (0.0)		0.12 (0.47)	0–2
I had to take care of a significant other who lost their home	58 (84.1)	5 (7.2)	6 (8.7)		0.25 (0.60)	0–2

### Relationship between flood-related trauma and post-flood mental health outcomes

The findings from the mental health correlation analyses are presented in Table 5. Damage to belongings was correlated with post-flood emotional distress (*r* = 0.238, *p* < 0.05). Loss of infrastructure was correlated with post-flood emotional distress (*r* = 0.339, *p* < 0.001) and post-flood anxiety (*r* = 0.321, *p* < 0.001). Interpersonal impact of floods was not correlated with any post-flood mental health outcomes.

**Table 5:** Correlation coefficients representing the relationship between impact trauma and pre- and post-flood mental health outcomes

	1	2	3	4	5	6	7
1. Damage to belongings	1						
2. Loss of infrastructure	0.569**	1					
3. Interpersonal impact	0.208	0.352**	1				
4. Emotional distress post-flood	0.238*	0.339**	0.089	1			
5. Anxiety post-flood	0.156	0.321**	0.224	0.718**	1		
6. Depression post-flood	0.054	0.199	0.041	0.612**	0.614**	1	
7. PTSD post-flood	0.189	0.088	0.055	0.649**	0.5819**	0.592**	1

*PTSD* Posttraumatic stress disorder

*p < 0.05, **p < 0.001

The findings from the regression analyses are presented in Table 6. The regression models were adjusted for baseline age and the baseline mental health measure corresponding with the post-flood mental health measure included in each model. The models estimating the effect of damage to belongings, loss of infrastructure and interpersonal impact of the floods on depression and PTSD were not significant. The models estimating the effect of damage to belongings and interpersonal impact of the floods on emotional distress and anxiety were also not significant. Loss of infrastructure related to the floods was significantly associated with emotional distress *ß* = 0.37, *t* = 2.10, *p* = 0.040 and anxiety *ß* = 0.40, *t* = 2.11, *p* = 0.039.

**Table 6:** Parameters for multiple regression models predicting mental health outcomes

	*ß*	std. error	Std. *ß*	*t*	*p*	95 CI	
						Lower	Upper
Emotional distress (model 1, *n* = 67)							
Damage to belongings	0.48	0.34	0.15	1.41	0.164	−0.20	1.15
Loss of infrastructure	0.37	0.17	0.22	2.10	0.040*	0.30	0.73
Interpersonal impact	0.02	0.24	0.01	0.08	0.938	−0.47	0.50
Depression (model 2, *n* = 65)							
Damage to belongings	0.24	0.85	0.03	0.28	0.779	−1.46	1.94
Loss of infrastructure	0.55	0.43	0.15	1.30	0.198	−0.30	1.40
Interpersonal impact	0.34	0.59	0.07	0.57	0.568	−0.84	1.51
Anxiety (model 3, 69)							
Damage to belongings	0.33	0.35	0.11	0.95	0.346	−0.37	1.04
Loss of infratructure	0.40	0.18	0.23	2.11	0.039*	0.020	0.76
Interpersonal impact	0.37	0.25	0.16	1.48	0.144	−0.13	0.87
PTSD (model 4, *n* = 68)							
Damage to belongings	1.13	0.72	0.15	1.56	0.124	−0.32	2.57
Loss of infrastructure	0.04	0.39	0.01	0.10	0.919	−0.74	0.82
Interpersonal impact	−0.22	0.53	−0.04	−0.41	0.682	−1.28	0.85

*PTSD* Posttraumatic stress disorder

*p < 0.05

### Relationship between food insecurity, prior trauma and pre- and post-flood mental health outcomes

The findings from the food security and prior trauma correlation analyses are presented in Table 7. Food insecurity was correlated with pre- (*r* = 0.339, *p* < 0.001) and post-flood (*r* = 0.356, *p* < 0.001) emotional distress, pre- (*r* = 0.451, *p* < 0.001) and post-flood (*r* = 0.329, *p* < 0.001) anxiety, pre- (*r* = 0.482, *p* < 0.001) and post-flood (*r* = 0.269, *p* < 0.05) depression, and pre-flood (*r* = 0.321, *p* < 0.001) PTSD. The composite pre-flood trauma score was correlated with pre- (*r* = 0.331, *p* < 0.001) and post-flood (*r* = 0.302, *p* < 0.001) emotional distress, pre- (*r* = 0.313, *p* < 0.05) and post-flood (*r* = 0.349, *p* < 0.001) anxiety, pre-flood depression (*r* = 0.262, *p* < 0.05) and pre- (*r* = 0.409, *p* < 0.001) and post-flood (*r* = 0.291, *p* < 0.001) PTSD.

**Table 7:** Correlation coefficients representing the relationship between food insecurity, prior trauma and mental health outcomes

	1	2	3	4	5	6	7	8	9	10
1. Food insecurity	1									
2. Prior trauma	0.209	1								
3. Emotional distress pre-flood	0.336**	0.331**	1							
4. Emotional distress post-flood	0.356**	0.302*	0.553**	1						
5. Anxiety pre-flood	0.451**	0.313**	0.689**	0.538**	1					
6. Anxiety post-flood	0.329**	0.349**	0.373**	0.718**	0.434**	1				
7. Depression pre-flood	0.482**	0.262*	0.616**	0.534**	0.612**	0.315*	1			
8. Depression post-flood	0.269*	0.207	0.358**	0.612**	0.296*	0.614**	0.398**	1		
9. PTSD pre-flood	0.321**	0.409**	0.625**	0.521**	0.607**	0.285*	0.689**	0.356**	1	
10. PTSD post-flood	0.179	0.291*	0.472**	0.649**	0.376**	0.519**	0.426**	0.592**	0.591**	1

*PTSD* Posttraumatic stress disorder

*p < 0.05, **p < 0.001

### Pathways to post-flood emotional distress

**Fig. 1﻿:**
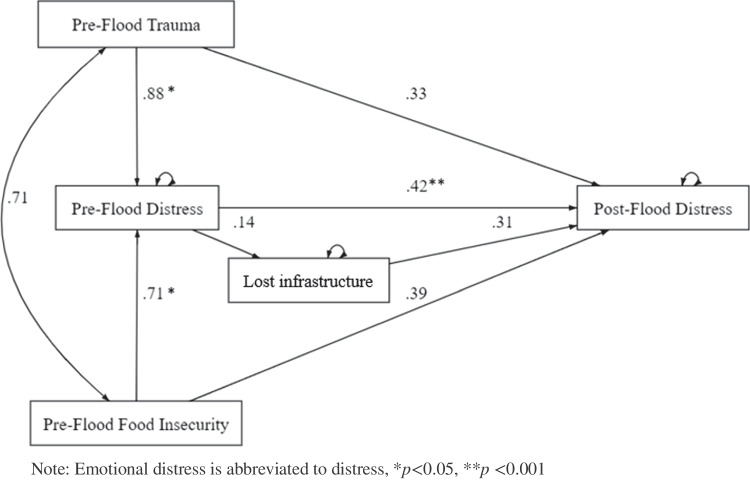
Pathways to post-flood emotional distress

The results for the structural equation model pathways to post-flood emotional distress is presented in Fig. 1 and Table 8. There was a direct path from pre-flood trauma (*ß* = 0.88, *p* = 0.014) and pre-flood food insecurity (*ß* = 0.71, *p* = 0.012) to pre-flood emotional distress. Increased pre-flood trauma and food insecurity was associated with increased pre-flood emotional distress. There was also a direct path from pre-flood emotional distress to post-flood emotional distress (*ß* = 0.42, *p* < 0.000) with higher pre-flood emotional distress predicting higher post-flood emotional distress. The association between increased loss of imfrastructure and increased post-flood emotional distress showed a trend towards significance (*ß* = 0.31, *p* = 0.060). There was an indirect path from pre-flood trauma to post-flood emotional distress (*ß* = 0.37, *p* = 0.037) and from pre-flood food insecurity to post-flood emotional distress (*ß* = 0.30, *p* = 0.034), with the direction of effect indicating that when mediated by increased pre-flood emotional distress, higher levels of pre-flood trauma and pre-flood food insecurity was likely to result in increased post-flood emotional distress.

**Table 8:** Loss of infrastructure and equation-level goodness of fit for the emotional distress SEM model

	Unstandardised						Standardised					
	B	SE	*z*	*p*	CIL	CIU	B	SE	*z*	*p*	CIL	CIU
Paths												
Pre-flood trauma → Pre-flood emotional distress	0.88	0.36	2.46	0.014*	0.18	1.58	0.28	0.11	2.6	0.011*	0.06	0.49
Pre-flood food insecurity → Pre-flood emotional distress	0.71	0.28	2.51	0.012*	0.16	1.26	0.28	0.11	2.61	0.009**	0.07	0.49
Pre-flood trauma → Post-flood emotional distress	0.33	0.33	1.01	0.314	−0.31	0.97	0.10	0.10	1.01	0.313	−0.10	0.31
Pre-flood food insecurity → Post-flood emotional distress	0.39	0.26	1.50	0.132	−0.12	0.90	0.16	0.10	1.51	0.132	−0.05	0.36
Pre-flood emotional distress → Post-flood emotional distress	0.42	0.11	3.92	< 0.001**	0.21	0.63	0.42	0.10	4.23	< 0.001**	0.23	0.62
Pre-flood emotional distress → Loss of infrastructure	0.14	0.07	2.02	0.044*	0.00	0.28	0.24	0.12	2.08	0.038*	0.01	0.46
Loss of infrastructure → Post-flood emotional distress	0.31	0.17	1.88	0.060	−0.014	0.64	0.19	0.10	1.89	0.059	−0.01	0.39
Variances												
Pre-flood trauma	2.86	0.49			2.04	4.02	1	0			1	1
Pre-flood food insecurity	4.65	0.80			3.31	6.52	1	0			1	1
Pre-flood emotional distress	23.62	4.08			16.84	33.14	0.81	0.09			0.66	1.00
Loss of infrastructure	9.80	1.69			6.99	13.75	0.94	0.06			0.84	1.06
Post-flood emotional distress	17.80	3.07			12.68	24.97	0.62	0.09			0.47	0.83
Equation-level goodness of fit	*R*^*2*^											
Pre-flood emotional distress	0.19											
Post-flood emotional distress	0.38											
Loss of infrastructure	0.06											

* *p *< .05, **p < .001

### Pathways to post-flood anxiety

**Fig. 2:**
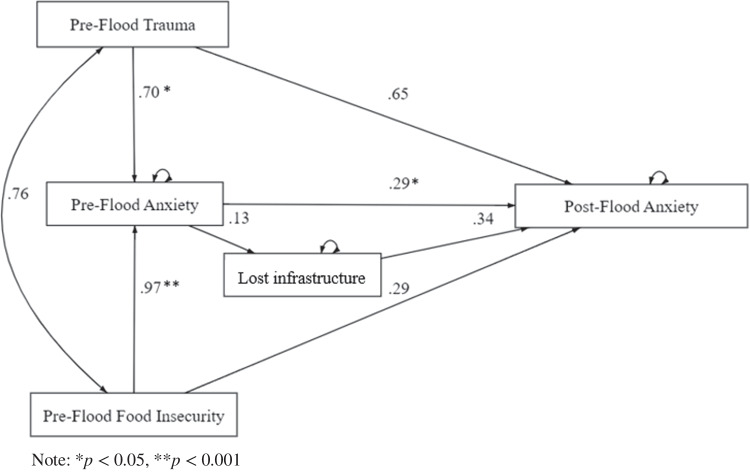
Pathways to post-flood anxiety. Note: Loss of infrastructure was shortened to lost infrastructure

The results for the structural equation model pathways to post-flood anxiety are presented in Fig. 2 and Table 9. There was a direct path from pre-flood trauma (*ß* = 0.70, *p* = 0.032) and pre-flood food insecurity (*ß* = 0.97, *p* < 0.000) to pre-flood anxiety. Increased pre-flood trauma and food insecurity were associated with higher pre-flood anxiety levels. The association between pre-flood trauma and post-flood anxiety showed a trend towards significance (*ß* = 0.65, *p* = 0.056). Higher pre-flood anxiety was associated with higher post-flood anxiety (*ß* = 0.29, *p* = 0.020) and the association between the higher score for loss of infrastructure and increased post-flood anxiety showed a trend towards significance (*ß* = 0.13, *p* = 0.064). There was an indirect path from pre-flood food insecurity to post-flood anxiety, with the direction of effect indicating that when mediated by increased pre-flood anxiety, higher levels of pre-flood food insecurity were likely to result in increased post-flood anxiety (*ß* = 0.28, *p* = 0.047).

**Table 9:** Loss of infrastructures and equation-level goodness of fit for the anxiety SEM model

	Unstandardised						Standardised					
	B	SE	*z*	*P*	CIL	CIU	B	SE	*z*	*p*	CIL	CIU
Paths												
Pre-flood trauma → Pre-flood anxiety	0.0.70	0.32	2.14	0.032*	0.06	1.33	0.23	0.10	2.19	0.029	0.02	0.43
Pre-flood food insecurity → Pre-flood anxiety	0.97	0.26	3.79	0.000**	0.47	1.47	0.40	0.10	4.10	0.000	0.21	0.60
Pre-flood trauma → Post-flood anxiety	0.65	0.34	1.91	0.056	−0.02	1.32	0.21	0.11	1.93	0.053	−0.00	0.42
Pre-flood food insecurity → Post-flood anxiety	0.29	0.29	1.00	0.318	−0.28	0.85	0.12	0.12	1.00	0.317	−0.11	0.34
Pre-flood anxiety → Post-flood anxiety	0.29	0.12	2.33	0.020*	0.05	0.53	0.28	0.12	2.40	0.02	0.05	0.50
Loss of infrastructure → Post-flood anxiety	0.34	0.18	1.89	0.058	−0.01	0.69	0.20	0.11	1.91	0.056	−0.01	0.41
Pre-flood anxiety → Loss of infrastructure	0.13	0.07	1.85	0.064	−0.01	0.28	0.22	0.11	1.90	0.058	−0.01	0.44
Variances												
Pre-flood trauma	2.84	0.48			2.04	3.97	1	0			1	1
Pre-flood food insecurity	4.59	0.78			3.29	6.41	1	0			1	1
Pre-flood anxiety	19.73	3.36			14.13	27.54	19.73	3.36			14.13	27.54
Post-flood anxiety	20.40	3.47			14.61	28.48	0.72	0.09			0.56	0.92
Loss of infrastructure	9.64	1.64			6.90	13.45	0.95	0.05			0.86	1.06
Equation-level goodness of fit	*R*^*2*^											
Pre-flood anxiety	0.25											
Post-flood anxiety	0.28											
Loss of infrastructure	0.05											

* *p *< .05, **p < .001

## Discussion

The findings of the study illustrate that the majority of women were affected by the floods with more than half reporting some damage to furniture, some damage to house structures, and lacking access to drinking water and electricity, for at least one to seven days. Nearly half also reported lacking access to fresh food for at least one to seven days. Witnessing death, knowing someone who passed away and knowing someone who was still missing due to the floods was reported by 25% of participants and 12% reported being uprooted and having to stay in a shelter. The high level of damage, loss and interpersonal impact is indicative of the severity of the floods experienced in April 2022 and its far-reaching effect on the livelihood of women living in low-income settings.

The mean pre- to post-flood scores for emotional distress, anxiety and PTSD remained relatively stable although 20.3% and 19.1% of participants who previously scored below the clinical cut-off for anxiety and PTSD pre-floods, scored above the clinical cut-off post-floods, respectively. There was a decline in pre- to post-flood depression scores, but 16.9% of participants who scored below the clinical cut-off pre-flood scored above the cut-off post-floods. The pre- and post-flood cumulative prevalence for anxiety was 43.5%, 38.2% for PTSD and 58.5% for depression. Studies reporting on the mental health effects of floods generally report post-flood prevalence rates with few having a baseline pre-flood exposure measure (Cianconi et al. 2020). A retrospective study conducted in a flood prone informal settlement in Kenya found that 80.8% of participants affected by floods experienced an increased in emotional distress in the immediate aftermath of floods (Okaka and Odhiambo 2019). A study conducted one-year post flooding in England found a significantly higher percentage of anxiety (39% vs 6.5%), PTSD (51.4% vs 7.9%) and depression (29.7% vs 5.8%) in participants who were flood affected vs unaffected (Waite et al. 2017). Participants who had to seek shelter as a result of the flooding were 6–7 times more likely to experience emotional distress (Waite et al. 2017). The slightly higher prevalence rates reported in this study is likely due to the effect of time, since participants were interviewed 2–3 months post-flooding rather than 1-year post-flooding (Fernandez et al. 2015).

Damage to belongings (furniture, electric appliances, cell phones/computers, house structure) and interpersonal impact (significant other was injured, still missing or passed away, witnessed someone getting injured or passing away, had to take care of someone who lost their home) were not associated with mental health outcomes. Loss of infrastructure (lack of access to drinking water, electricity, fresh food, transport, cell phone signal and housing) was a predictor of post-flood emotional distress and anxiety while taking into account pre-flood emotional distress and anxiety scores. This finding is in line with prior findings indicating that those who are directly and more severely affected by floods are at higher risk of experiencing psychological distress compared to those who are indirectly affected (Bouchard et al. 2022; Waite et al. 2017). The short- to medium-term recovery from floods is generally characterized by a busy period of regaining access to primary needs e.g. rebuilding damaged property and infrastructure and seeking shelter, food and drinking water which likely explains why those who were more severely affected by the loss of infrastructure were experiencing higher levels of distress and anxiety (Bouchard et al. 2022). Mental health effects and recovery from flood-related trauma, and possibly those related to interpersonal impact (injury and death), is likely to occur in the longer-term and to result in psychological disorders such as depression and PTSD (Bouchard et al. 2022; Waite et al., 2017). Flood-related development of psychological disorders may develop at a later point in recovery e.g. some studies report that symptoms of PTSD peak around a year post-floods when seasonal changes results in heavy rainfall again (Assanangkornchai et al. 2006; Cianconi et al. 2020; Fernandez et al. 2015).

A composite score of pre-flood prior experiences of childhood trauma, IPV and NPSV was associated with pre-flood emotional distress and anxiety. Women reporting higher pre-flood trauma and higher pre-flood emotional distress were also more likely to report higher post-flood levels of emotional distress. It is likely that the floods exacerbated anxiety symptoms in women who were already experiencing high levels of anxiety prior to the floods, as has been observed in other studies investigating flood-exposed communities in LMIC (Bhamani et al. 2012; Fernandez et al. 2015; Stanke et al. 2012). Prior experiences of trauma, especially those occurring during childhood may result in long-term changes in biological and psycho-social response to stress which may leave an individual vulnerable to adverse mental outcomes when trauma is compounded with additional events such as flooding (Zarse et al. 2019).

Higher levels of pre-flood food insecurity were correlated with higher levels of pre-flood emotional distress and anxiety. Women reporting higher pre-flood food insecurity were also more likely to report higher post-flood anxiety. Lack of access to resources have been linked to adverse mental health outcomes following flooding in previous studies, especially in the context of poverty, loss of belongings and loss of shelter (Bouchard et al. 2022; Cianconi et al. 2020). The severe damage to infrastructure observed in the April floods resulted in many women being unable to travel, work and collect wages and food insecure children were unable to access school feeding schemes (Bouchard et al. 2022). The anxiety associated with being unable to adequately feed children and family members was most likely compounded by the extra flood-related barriers to meeting these needs (Cianconi et al. 2020).

### Strengths and limitations

A particular strength of the study is that a baseline mental health assessment was available for this sample of women before the floods occurred which allowed us to compare pre- to post-flood changes in mental health. However, the sample was small and limited to women from low-income settings in the eThekwini municipal region of Durban, which limits generalizability to males and those residing in higher income regions who were also affected by the floods. The use of convenience sampling further limits generalizability. The flood impact questionnaire and the composite trauma load score has not been used before in prior studies and has not undergone a comprehensive validation assessment. We did not assess the availability and use of material and mental health assistance in the post-flood period and it is likely that these forms of assistance may have played a protective role against adverse mental health outcomes. Similarly, we also did not investigate physical health, family- and intimate relationship-related variables which may have served as risk or protective factors. The assessments were completed within two to three months of the floods occurring and the potential longer term adverse mental health effects related to floods was not captured.

### Conclusion

In conclusion, rapid expansion of informal settlements coupled with insufficient infrastructure development (e.g. water, sanitation, storm water drains), service planning and delivery (e.g. access to food, shelter, emergency response, healthcare) and high levels of poverty potentially places those exposed to floods at higher risk for adverse mental health outcomes (Borg et al. 2021; Opoku et al. 2021). Proactive approaches to diminishing the impact of floods are needed such as improving and maintaining infrastructure and service delivery as well as creating flood warning systems that will reach informal settlements (e.g. community informants and social media alerts rather than formal weather forecasts disseminated through television and internet-based news channels) (Fernandez et al. 2015; Okaka and Odhiambo 2019). Women affected by poverty, food insecurity, a history of trauma and prior adverse mental health may be vulnerable to the additive adverse mental health effects of natural disasters such as floods, but it is also likely that women who are constantly faced with adversity have a higher tolerance to additive stressors and are able to recover more rapidly than what would be expected (Bouchard et al. 2022; Charlson et al. 2021; Fernandez et al. 2015). Longitudinal follow-up studies conducted in LMIC over a period of a year or more may provide a clearer picture of the acute, post-acute and chronic adverse mental health outcomes associated with floods.

### Supplementary Information

Below is the link to the electronic supplementary material.

Supplementary file1 (DOCX 22.7 KB)

## Data Availability

The data used in this paper are available on request to the corresponding author.
