# Supplementary material for: Change in emotional distress, anxiety, depression and PTSD from pre- to post-flood exposure in women residing in low-income settings in South Africa
Source: Arch Womens Ment Health. 2023 Nov 22;27(2):201–18. doi: 10.1007/s00737-023-01384-3 (PMC10933147; doi:10.1007/s00737-023-01384-3)
Supplement: Supplementary file 1 — Supplementary file1 (DOCX 22.7 KB) [file 737_2023_1384_MOESM1_ESM.docx]

Supplementary Table 1: *Baseline demographic, food insecurity, trauma and mental health characteristics of the sample*

|  | Pre-flood interview only  (n = 31) | | | Pre- and post-flood interview  (n = 69) | | |  |
| --- | --- | --- | --- | --- | --- | --- | --- |
|  | n (%) | M (SD) | Range | n (%) | M (SD) | Range | *p* |
|  |  |  |  |  |  |  |  |
| Age | 31 (100) | 24.3 (5.3) | 19-38 | 69 (100) | 26.3 (5.7) | 18-44 | .089 |
|  |  |  |  |  |  |  |  |
| Level of education completed | 31 (100) |  |  | 69 (100) |  |  | .776 |
| No formal education | 0 (0.0) |  |  | 0 (0.0) |  |  |  |
| Primary education | 19 (61.3) |  |  | 40 (58) |  |  |  |
| Secondary education | 23 (38.7) |  |  | 28 (40.6) |  |  |  |
| Tertiary education | 0 (0.0) |  |  | 1 (1.4) |  |  |  |
|  |  |  |  |  |  |  |  |
| Main source of income | 31 (100) |  |  | 69 (100) |  |  | .955 |
| No income | 0 (0.0) |  |  | 1 (1.4) |  |  |  |
| Employment | 4 (12.9) |  |  | 9 (13.4) |  |  |  |
| Social grant | 22 (71.0) |  |  | 49 (71.0) |  |  |  |
| Family support | 3 (9.7) |  |  | 8 (11.6) |  |  |  |
| Partner support | 2 (6.5) |  |  | 5 (7.2) |  |  |  |
| Other^1^ | 0 (0.0) |  |  | 1 (1.4) |  |  |  |
|  |  |  |  |  |  |  |  |
| Amount of money available to spend per month^2^ | 31 (100) |  |  | 68 (100) |  |  | .102 |
| None | 0 (0.0) |  |  | 0 (0.0) |  |  |  |
| R1 – R500 ($0 – $31) | 20 (64.5) |  |  | 29 (42) |  |  |  |
| R501 – R1000 ($31 – $61) | 5 (16.1) |  |  | 28 (30.6) |  |  |  |
| R1001 – R2000 ($61 - $122) | 5 (16.1) |  |  | 9 (13) |  |  |  |
| > R2001 (>$122) | 1 (3.2) |  |  | 2 (2.9) |  |  |  |
|  |  |  |  |  |  |  |  |
| Household Hunger Scale (HHS) score | 31 (100) | 4.5 (2.3) | 3-11 | 69 (100) | 4.6 (2.2) | 3-12 | .871 |
| Childhood Trauma Scale (CTQ) score | 29 (100) | 22.2 (5.1) | 16-36 | 69 (100) | 22.2 (6.5) | 14-40 | .885 |
| Experienced childhood neglect | 19 (63.3) |  |  | 38 (55.1) |  |  |  |
| Experienced childhood emotional abuse | 14 (45.2) |  |  | 36 (52.2) |  |  |  |
| Experienced childhood physical abuse | 23 (74.2) |  |  | 48 (69.6) |  |  |  |
| Experienced childhood sexual abuse | 8 (25.8) |  |  | 20 (29.0) |  |  |  |
| Intimate partner violence (IPV) | 31 (100) |  |  | 69 (100) |  |  |  |
| Experienced physical IPV | 9 (29.0) |  |  | 24 (34.8) |  |  |  |
| Experienced sexual IPV | 11 (35.5) |  |  | 24 (34.8) |  |  |  |
| Non-partner violence (NPV) | 31 (100) |  |  | 69 (100) |  |  |  |
| Experienced sexual NPV | 6 (19.4) |  |  | 10 (14.5) |  |  |  |
| Composite pre-flood trauma load score | 30 (100) | 2.9 (1.7) | 1-7 | 69 (100) | 2.9 (1.7) | 0-7 |  |
| Emotional distress (K6) pre-flood | 31 (100) | 15.8 (5.3) | 16-58 | 67 (100) | 15.9(5.4) | 6-24 | .960 |
| Depression (CESD) pre-flood | 31 (100) | 24.7 (12.5) | 1-51 | 66(100) | 23.9(12.4) | 3-51 | .750 |
| Anxiety (GAD7) pre-flood | 29 (100) | 8.5 (5.4) | 0-21 | 69(100) | 8.4(5.2) | 0-21 | .957 |
| PTSD (HTQ) pre-flood | 29 (100) | 33.3 (10.7) | 16-58 | 68(100) | 32.9(13.1) | 16-63 | .885 |

1. Student bursary funding provided by the South African National Student Financial Aid Scheme (NSFAS)

2. Based on the average United States Dollar ($) to South African Rand (ZAR) exchange rate of $1 to R16.4 in 2022

Abbreviations: Household Hunger Scale (HHS), Childhood Trauma Scale (CTQ), intimate partner violence (IPV), non-partner violence (NPV), Kessler Psychological Distress Scale (K6), Centre for Epidemiologic Studies Depression Scale (CES-D), Generalised Anxiety Disorder 7 (GAD7), posttraumatic stress disorder (PTSD), Harvard Trauma Questionnaire (HTQ).
